# Enrichment of CD44 in Exosomes From Breast Cancer Cells Treated With Doxorubicin Promotes Chemoresistance

**DOI:** 10.3389/fonc.2020.00960

**Published:** 2020-07-14

**Authors:** Xiaohong Wang, Kai Cheng, Guoqiang Zhang, Zhongming Jia, Yue Yu, Jiwei Guo, Yitong Hua, Fengli Guo, Xiaoqiang Li, Weiwei Zou, Hongguang Sun, Jianli Dong, Zhenlin Yang

**Affiliations:** Department of Thyroid and Breast Surgery, Binzhou Medical University Hospital, Binzhou, China

**Keywords:** breast cancer, chemoresistance, CD44, exosomes, proteomics

## Abstract

Exosomes secreted from tumor cells can remodel the tumor environment by promoting tumor metastasis and multidrug resistance. The aim of this study was to analyze the proteome profile of the breast cancer line resistant to doxorubicin resistance (MCF-7/ADR) by liquid chromatography linked to tandem mass spectrometry assay (LC-MS/MS). Our results revealed that DOX increases the exosomes release from MCF-7/ADR cells and the exosome-mediated proteins intercellular transfer in breast cancer chemoresistance regulation. The expression of the candidate target exosomic CD44 in DOX-resistant cells (A/Exo) was higher than in parental breast cancer cells (S/Exo), and the increasing levels of exosomic CD44 (21.65-fold) were higher than those of cellular CD44 (6.55-fold) (all *p* < 0.05). Similar results were obtained in clinical samples; exosomal CD44 in the serum of nonresponders was significantly higher than that in the chemotherapy-responsive group (*p* < 0.05). Also, we modified the MCF-7–derived exosomes loaded with siRNA against CD44 to observe the effects of targeting reduced CD44 expression in luminal A breast cancer cells. Exosome-siRNA targeted CD44 (Exos-siCD44) could efficiently silence its expression. When cocultured on Exos-siCD44, breast cancer cells exhibited reduced cell proliferation and enhanced susceptibility to DOX. The same phenomenon was observed in mice. In conclusion, breast cancer cells could spread resistance capacity by the intercellular transfer of proteins, especially CD44, via exosomes.

## Introduction

Breast cancer is the most common cancer in females. Doxorubicin (DOX) is a well-known thracycline antibiotic commonly used to treat patients with breast cancer. However, owing to acquired drug resistance, the response rate of DOX therapy is still relatively low (25–40%) (1). Multidrug resistance analysis of breast cancer is of great importance for predicting the effect and outcome of chemotherapy and overcoming the barrier of drug resistance. Proteomics comparing analyses between drug-resistant and drug-sensitive breast cancer cells have provided a good foundation for understanding the development of drug resistance (2–5). So far, several resistance-related proteins have been identified in breast cancer cells (6); yet, little is known about the transmission pattern of drug resistance information between the sensitive and resistant breast cancer cells.

Exosomes are membranous nanovesicles (30–150 nm size) that transport active cargoes between different cells and can be found in many bodily fluids. Their cargo transports disease-specific molecular information and can reflect the real-time physiological status of the disease, including tumors (7). Over recent years, the role of exosomes in inducing chemoresistance has gained increasing research interest (8). Exosome signaling creates a favorable condition for tumor microenvironment and therapy strategy for tumor heterogeneity (9, 10). In our previous study, we found that the levels of exosomes released from DOX-resistant breast cancer cells (MCF-7/ADR) are higher compared to those in nonresistant cells, and their transcriptome changes in response to DOX (11). Mechanically, the exosomes mediate drug resistance among cells in the following ways: direct drug export, drug efflux pumps transportation, and miRNAs exchange (12). However, the protein cargo and function transmitted by exosomes in response to DOX remain unclear. Proteomics analysis may provide a better understanding of the role of exosomes in intercellular chemoresistance transfer.

The aim of this study was to analyze the proteome profile of the breast cancer line MCF-7 in dependence on DOX resistance by liquid chromatography linked to tandem mass spectrometry assay (LC-MS/MS). Our results may be used to develop a potential therapy for chemotherapy resistance in breast cancer.

## Materials and Methods

### Cell Culture

The MCF-7 cell lines was purchased from Nanjing KeyGen. MCF-7/ADR cells were established through DOX gradient concentration. MCF-7 cells were cultured in RPMI-1640 medium supplemented with 10% exosome-depleted fetal bovine serum (FBS). MCF-7/ADR were cultured in the medium with the final concentration of 1 μg/mL of DOX to maintain the drug-resistant phenotype. All cells were tested negative for mycoplasma and maintained in 5% CO_2_ at 37°C.

### Exosome Isolation, Identification, and Peptide Extraction

To measure the participation of exosomes in the DOX-induced transmission of drug resistance, sequential ultracentrifugation was used to isolate exosomes from MCF-7 and MCF-7/ADR cells that were cultured in the exosome-depleted medium for 48 h, as previously described (11). The exosome-depleted medium was prepared as follows: medium was ultracentrifuged at 120,000 *g* for 16 h and then filtered using a 200-nm filter. Exosomes were identified by transmission electron microscopy (TEM) and ZetaView nanoparticle-tracking analysis (NTA) instrumentation.

The exosome-specific markers were detected by western blot analysis. Briefly, exosomes were lysed with radioimmunoprecipitation assay (RIPA) buffer. The obtained proteins were quantified using NanoDrop (Thermo Fisher Scientific, Waltham, MA, USA) and analyzed by sodium dodecyl sulfate-polyacrylamide gel electrophoresis (SDS-PAGE) and detected by Odyssey® Infrared Imaging System (LI-COR). The incubation concentration was 1:100 for anti-CD63 (Proteintech Group) and anti-calnexin antibodies (Proteintech), followed by IRDye® 800CW IgG (H+L) and IRDye® 680RD IgG (H+L) antibody (LI-COR, 1:15,000). Protein separation, digestion, and peptide extraction for LC-MS/MS were prepared according to the previously described methods (13). The peptides obtained were labeled by Tandem Mass Tag™ (TMT™, Thermo Fisher Scientific, Waltham, MA, USA) and cleaned, desalted, and vacuum dried.

### Quantitative Proteomic Analysis and Bioinformatic Analysis

DOX-resistant (A/Exo) and parental breast cancer cells (S/Exo) were analyzed with an online two-dimensional nano LC/MS/MS by BioNovoGene. The differently expressed proteins (DEPs) between S/Exo and A/Exo were selected by the different multiples (log2FoldChange|?1.0) and significant levels (*q*-value < 0.001). The gene ontology analysis (GO) enrichment analysis and Kyoto encyclopedia of genes and genomes (KEGG) pathway enrichment analysis were then performed.

### Flow Cytometry Analysis to Verify the LC-MS/MS Results

Considering that the amount of protein in exosomes is low, flow cytometry analysis was selected. Briefly, exosomes were resuspended in 100 μL of phosphate-buffered saline (PBS) and attached to 10 μL of aldehyde/sulfate latex beads (4 μm, A37304, Invitrogen, Carlsbad, CA, USA) for 2 h of continuous rotation at room temperature, and consequently mixed with 1 mL of PBS. The samples continued to rotate for 2 h at room temperature, after which they were centrifuged at 580 g for 5 min to collect pellet. Then, the samples were incubated in 1 mL of 100 mM glycine solution for 30 min. Exosome-bounded beads were washed by 1× PBS/2% bovine serum albumin (BSA) and centrifuged at 14,800 *g* for 1 min, blocked with 10% BSA with rotation for 30 min, washed, and centrifuged. Consequently, exosome-bounded beads were incubated with the respective antibodies (listed in Table S4) at 4°C for 30 min with continuous rotation. Isotype Control incubation was used to gate the beads with CD63+ and respective antibody-bounded exosomes, respectively. The percent of positive beads was calculated in relation to the total number of CD63+ beads analyzed per sample; finally, the log2 value was obtained.

### Exosomal CD44 of Breast Cancer Cells Were Detected by Western Blotting and Immunogold Labeling

To further validate the different levels of CD44 in S/Exo and A/Exo, we performed immunogold labeling and western blot analysis, which were conducted according to the previous protocol. Exosomes were fixed in 1% paraformaldehyde with 0.05% glutaraldehyde. Samples were then incubated with the antibody of CD44 (Proteintech Group, Rosemont, IL, USA) at a 1:30 dilution for 36 h at 4°C; the isotype-matched antibody served as a negative control. Then all of the grids were floated on drops of 6-nm gold particles (1:30 dilution) for 1 h at room temperature. Samples were observed under a transmission microscope after negative staining with filtered aqueous 2% uranyl acetate for 1 min. Excess uranyl acetate was drained with filter paper, and samples were examined on TEM at an accelerating voltage of 120 kilovolts (kV). Western blot analysis was performed according to the previous protocol after protein quantification. Signals were visualized and quantitatively analyzed with Odyssey Sa Quantitative Infrared Imaging System (LI-COR Biosciences, Lincoln, NE, USA). β-Actin was used as an internal reference for cells, while CD63 (Proteintech Group, Rosemont, IL, USA) was used as the internal reference for exosomes.

### Effect on MCF-7 After Incubation With ADR/Exo

To evaluate the changes of CD44 in MCF-7 after incubation with ADR/Exo, 1 × 10^6^ MCF-7 cells were cultured in six-well plates with different concentration (1 × 10^5^, 1 × 10^6^, 1 × 10^7^, 1 × 10^8^, and 1 × 10^9^ particles/mL incubation for 7 days) and different time (1 × 10^8^ particles/mL ADR/Exo harvested at days 0, 1, 3, 5, and 7) continuous passage cultures and added every other day. Cells were respectively harvested and stained with allophycocyanin (APC)-anti-human CD44 antibody at room temperature for 30 min (BioLegend, San Diego, CA, USA, CN. 338806, 4 μL of antibody in 100 μL of 1× PBS). APC-labeled IgG1κisotype controls (BioLegend, San Diego, CA, USA, CN. 3400119) were used as negative controls. CD44 expression in MCF-7, MCF-7/ADR, and MCF-7+ADR/Exo cell lines was assayed by flow cytometry analysis. According to the best time and concentration obtained, we further examined the DOX sensitivity of MCF-7 after 7 days of incubation with 1 × 10^8^ particles/mL ADR/Exo by Cell Counting Kit-8 (CCK-8).

### Flow Cytometry Analysis of the Levels of Exosomal CD44 Levels in Patient Plasma

To validate the exosomal CD44 as a diagnostic and predictive tool of response to DOX, peripheral blood samples were obtained from 54 stage IIa–IIIc luminal A breast cancer patients who received neoadjuvant chemotherapy with epirubicin (75 mg/m^2^, q21d) plus docetaxel (75 mg/m^2^, q21d) on day 1 from January 2019 to December 2019. Clinical data of the patients were also collected. The tumor response was evaluated by residual tumor burden, which was measured by magnetic resonance imaging (MRI). The pathologic response was assessed according to the Response Evaluation Criteria in Solid Tumors (RECIST) criteria as stable disease (SD), progressive disease (PD), partial response (PR), or complete response (CR). CR was defined as disappearance of all target lesions; PR, a minimum of 30% decrease in the sum of the diameters of the target lesions; PD, a minimum of 20% increase in the sum of the diameters of the target lesions; SD, insufficient shrinkage to qualify as PR and insufficient increase to qualify as PD. The sizes of the target lesions were based on the MRI data. Patients achieving a CR or PR (PR/CR) were considered chemotherapy responders. Nonresponders were categorized as either SD or PD (PD/SD).

The study was approved by the Chinese Clinical Trials Registry (Registration number is ChiCTR1900026195), and all patients gave written informed consent (ethical vote number LW-012). The plasma samples were collected from 28 patients who responded to chemotherapy (15 PR and 13 CR) and 26 patients who did not (11 PD and 15 SD). All blood samples were taken before treatment and centrifuged at 2500 *g* for 10 min to extract the plasma. The plasma was stored at −80°C until analyzed. Exosomes were isolated from human serum samples according to the protocol described before. Briefly, 250 μL of cell-free serum was added to 11 mL 1× PBS. After filtration (0.2 μm pore filter), the samples were ultracentrifuged at 120,000 *g* at 4°C for 6 h. Next, the exosomes pellet was washed in 11 mL of 1× PBS. After centrifugation, the collected exosomes were used for immunoelectron microscopy and flow cytometry analysis according to the previous research (14). Exosome-bounded beads were incubated with CD63 Monoclonal Antibody (H5C6)-Phycoerythrin (PE) (Thermo Fisher Scientific, CN.12-0639-41) and APC anti-human CD44 Antibody (BioLegend, CN. 338806) at 4°C for 30 min with continuous rotation. Isotype Control incubation was used to gate the beads with CD63+, and CD44+ bounded exosomes, respectively. The percent with CD44 positive beads were calculated relative to the total number of beads analyzed per sample. A paired two-tailed Student's *t*-test was used to analyze the differences of CD44+ exosomes between patients who responded to chemotherapy and those who did not.

### Enzyme-Linked Immunosorbent Assay

For further detection of CD44 of exosomes and patients' plasma, ELISA plates (96-well) (BioLegend) were coated with 0.25 μg of CD44 antibody at 4°C for 12 h. Free binding sites were blocked, and 100 μL of plasma samples without exosome removal or exosome samples purified from plasma were added to each well to analyze the concentration of CD44. Receiver operating characteristic curves (ROC) were used to analyze the sensitivity, specificity, positive, and negative predictive value.

### Construction of iRGD Exosomes

To acquire modified exosomes that can target breast cancer cells, iRGD exosomes were established. First, the iRGD peptide was cloned into lysosome-associated membrane glycoprotein 2b (Lamp2b). MCF-7 cells were transfected with plasmid to harvest iRGD exosomes using Lipofectamine 3000 transfection reagent, according to the manufacturer's protocol (Invitrogen, Carlsbad, CA, USA). The medium was changed 6h after transfection. The cells were then incubated at 37°C for 48 h to harvest the medium for density gradient centrifugation and NTA analysis, as described earlier, so as to obtain the iRGD-Exosomes. The iRGD-Lamp2b mRNA expression in the MCF-7 cells was detected using real-time-polymerase chain reaction (RT-PCR) at 48 h after transfection.

### siRNA-CD44 Encapsulation Into Exosomes by Electroporation

The electroporation cuvette was prechilled for 30 min before electroporation. Then, 7.0 μg of exosomes was mixed with 0.33 μg of siRNA in the microcentrifuge tube with citric acid buffer reaching the final volume of 150 μL (15). TEM was then used to analyze the exosome–siRNA complex at different ratios with zeta potential and their structure; a 1:60 ratio had the highest transfection efficiency at the lowest possible cytotoxicity. The mixture was then transferred to an electroporation cuvette, and the free siRNA was removed using size-exclusion chromatography. Finally, the iRGD-Exos-siRNA/CD44 (Exos-siCD44) was obtained. The siRNA-CD44 sequence was GAAACTCCAGACCAGTTTA; negative control siRNA (siRNA-NC) sequence was TTCTCCGAACGTGTCACGT.

### Exosome Morphology Changes Analyzed by TEM and NTA

We harvested the cultural supernatants of MCF-7 cells transfected with iRGD-Exos-siRNA/CD44 for 48 h and obtained exosomes by centrifugation as described earlier. The exosome morphological integrity and size changes were analyzed by TEM and NTA. The collected exosomes were re-suspended in PBS for the following use.

### Exosome Labeling and Detection of Cell Uptake Efficiency

For cell uptake experiment, exosomes were stained with the anti-CD63-Alexa Fluor 488; and with lipophilic tracers 1,10-dioctadecyl-3,3,30,30 -tetramethylindodicarbocyanine (DiD; Invitrogen, Carlsbad, CA) for animal imaging experiments. For CD63 labeling, the CD63-Alexa Fluor 488 antibody was added at a concentration of 1:50 and incubated for 30 min at 37°C. A total of 4 × 10^8^, 4 × 10^9^, and 4 × 10^10^ exosomes were respectively incubated with DiD at the concentration of 10, 15, 20, and 25 μg/mL for 30 min at 37°C to determine the optimal incubation conditions. Then exosomes were centrifuged at 120,000 *g* for 90 min to remove the free dye and washed twice using PBS to get the labeled exosomes. To investigate whether iRGD-Exos can target MCF-7 cells, anti-CD63-Alexa Fluor 488 labeled exosomes were cultured with MCF-7 cells for 0.5, 2, 4, 8, and 24 h. The uptake efficiency was detected by a confocal laser-scanning microscope and analyzed through mean fluorescence intensity analysis (MFI).

### Role of iRGD-Exosomes-siRNA CD44 on Cell Proliferation and Drug Resistance in MCF-7/ADR Cells

The expression of CD44 in MCF-7/ADR cells after 48 h of incubation with Exos-siCD44 was detected by RT-PCR and western blot. The effects of silencing CD44 mediated by iRGD modified exosomes on DOX sensitivity of MCF-7/ADR cell were also measured by CCK-8 assay. Briefly, MCF-7/ADR cells were incubated with Exos-siCD44 and iRGD-exosomes-siRNA NC (Exos-NC), respectively. After 12 h, 2 × 10^4^ cells per well were cultured in 96-well plates overnight. The next day, 20 μL of CCK-8 was added to each well, and cells were incubated for 2 h. Absorbance values were then measured at 490 nm using a microplate reader (Thermos). The sensitivity of the cells to DOX was measured by calculating the IC_50_ values. The experiment was repeated three times, each time with three parallel samples.

### *In vivo* Bioluminescence Imaging and Anticancer Efficacy

Six-week-old female BALB/c nude mice were purchased from Beijing Vital River Laboratories. All the animals were housed in an environment with a temperature of 22 ± 1°C, relative humidity of 50 ± 1%, and a light/dark cycle of 12/12 h. All animal studies (including the mice euthanasia procedure) were done in compliance with the regulations and guidelines of Binzhou Medical University Hospital institutional animal care and conducted according to the AAALAC and the IACUC guidelines (No. SYXK 20180022).

A total of 1.0 × 10^7^ MCF-7/ADR cells in 50 μL of PBS mixed with 50 μL of Matrigel were transplanted into the second left fat pad to establish the animal model. The tumor volume was calculated through length × width^2^/2. When the tumor size reached 0.1 cm^3^, the mice were randomly divided into 8 groups (6 mice/group): (1) PBS, as a control; (2) Exo-NC; (3) Exo-siCD44; (4) Exo-siCD44 combined with weekly intraperitoneal DOX (4 mg/kg); (5) equivalent dose of intraperitoneal DOX; (6) equivalent dose of free siRNA/CD44; (7) blank-Exo group for biodistribution analysis (three for *in vivo* observation and three for tissue observation); (8) iRGD-Exo group for biodistribution analysis (three for *in vivo* observation and three for tissue observation). DiD-labeled S/Exo was administered by tail vein to study the biodistribution of exosomes. Biofluorescence images and the *ex vivo* images were obtained using the IVIS imaging system (PerkinElmer, Waltham, MA, USA). iRGD-Exo-siRNA/CD44 were injected twice with an interval of 3 days; tumor sizes were measured every 3 days. On the 28th day, the tumors were excised from sacrificed mice. To evaluate the expression of CD44, immunohistochemical staining was conducted using the excised tumors. Three different fields were randomly chosen for scoring by two pathologists in a blinded manner under the microscope (×200).

### Statistical Analysis

The GraphPad Prism version 8.0 was used for all calculations. The *t*-test and Analysis of Variance (ANOVA) were used to compare the mean between samples. ROC and the area under ROC (AUC) were used to determine the sensitivity, specificity, and positive and negative predictive value of exosomal CD44. A value of *p* < 0.05 was considered to be statistically significant.

## Results

### DOX Induces Exosome Release From MCF-7/ADR Cells

**Figure 1:**
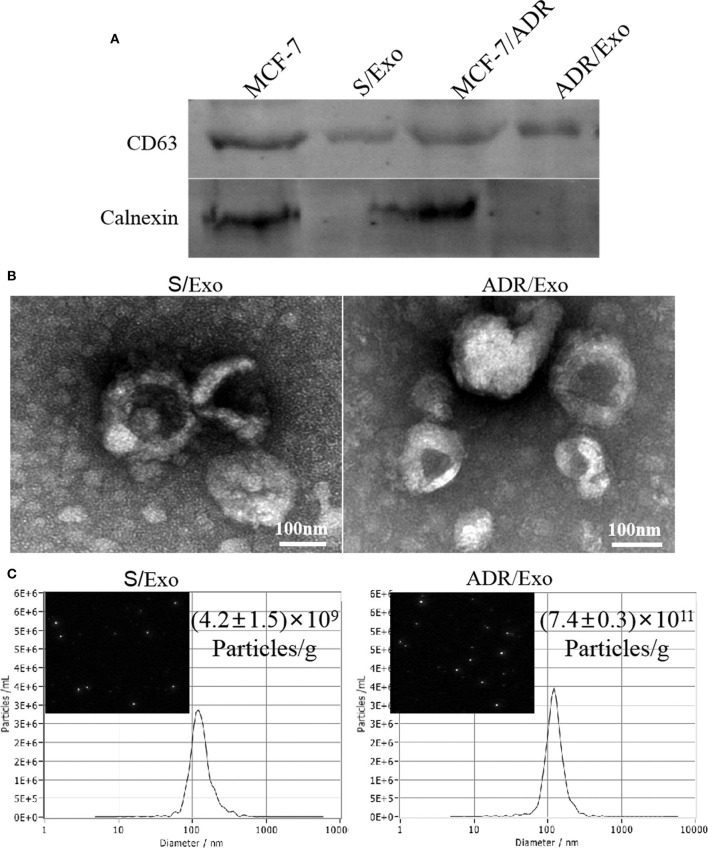
DOX increased the exosomes release of MCF-7/ADR cells. **(A)** Exosome identification by western blot. These vesicles expressed conventional exosomal markers CD63; calnexin was detected only in total cellular lysates and not in exosomes. **(B)** The morphology of exosomes was analyzed using TEM, showing a typical “saucer-like” morphology and a diameter of 30–120 nm (scale bar, 100 nm). **(C)** The size distribution and concentration of A/Exo and S/Exo were analyzed by NTA. The concentration of exosomes was (7.4 ± 0.3) × 10^11^ particles/g in A/Exo and (4.2 ± 1.5) × 10^9^ particles/g in S/Exo. There were no significant differences in morphology and size distribution between S/Exo and A/Exo (*p* > 0.05), while the concentration of A/Exo was significantly higher than that of S/Exo (*p* < 0.05).

We first examined the effect of chemotherapy (DOX) on the release of exosomes from cancer cells (MCF-7/ADR cells). Cells treated with DOX produced vesicles that expressed conventional exosomal markers CD63, while calnexin was detected only in total cellular lysates and not in exosomes (Figure 1A). Moreover, NTA and TEM revealed a size range of 121 ± 5 nm and 117 ± 4 nm in diameter, respectively (*p* > 0.05; Figures 1B,C). Both exosomes (A/Exo and S/Exo) revealed similar size distributions and morphologies. The concentration of exosomes was (7.4 ± 0.3) × 1011 particles/g in A/Exo and (4.2 ± 1.5) × 109 particles/g in S/Exo (*p* < 0.05) when analyzing the same volume (Figure 1C). Our results showed that treatment with DOX increased the exosomes' release of MCF-7/ADR cells.

### Proteomic Analysis and Bioinformatic Analysis

**Figure 2:**
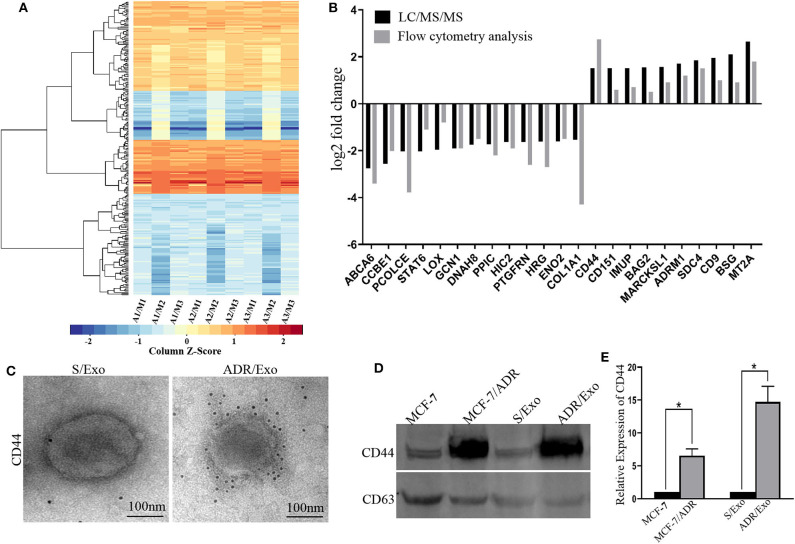
DOX-resistant breast cancer cells spread resistance capacity by transferring resistance-associated proteins, especially CD44, to sensitive cells. **(A)** The exosomes extracted from MCF-7/ADR cells with three biological replications were labeled as A1, A2, and A3. The exosomes extracted from MCF-7 cells with three biological replications were labeled as M1, M2, and M3. A/M stands for the log2 (fold change) results, which was the average protein signal ratio of the A and M groups. Then we performed the heat map analysis based on the log2 (fold change) results. In the heat map analysis, blue indicates the downregulated proteins, and red indicates the upregulated. **(B)** We further verified these differently expressed proteins by flow cytometry analysis, which was also consistent with the proteomic analysis. **(C)** The immunogold staining showed that more immunogold particles were bound to the exosomes, thus? indicating that CD44 was highly stored inside A/Exo. **(D,E)** Western blot results indicated that the protein level of CD44 was significantly upregulated in A/Exo compared with that in S/Exo (*p* < 0.05), and the increase level of CD44 in A/Exo (14.76-fold) was much higher than that in MCF-7/ADR cells (6.56-fold). *p < 0.05.

The induced resistance capacity of chemotherapy-elicited exosomes may depend on their protein repertoire. Thus, we performed a proteomic analysis of DOX-resistant (A/Exo) and parental breast cancer cells (S/Exo) using LC-MS/MS. In comparison with S/Exo, A/Exo showed 250 different proteins, including 128 downregulated proteins and 122 upregulated proteins (Figure 2A and Tables S1, S2). These were further confirmed using flow cytometry analysis (Figure 2B).

To investigate the functional basis of the identified chemotherapy-resistant proteins, GO analysis revealed that the differently expressed proteins mainly regulate cell–cell adhesion, cell–cell adherens junction, extracellular matrix organization, extracellular region, and cadherin binding involved in cell–cell adhesion (Figure S1). KEGG analysis showed that the different proteins were mainly involved in the extracellular matrix–receptor interaction, focal adhesion, complement and coagulation cascades, plate activation, and PPAR signaling pathway (Table S3).

### DOX-Elicited Exosomes Are Enriched in CD44

CD44 has an important role in mediating chemoresistance and cell proliferation (16). In this study, we investigated the role of exosomic CD44 in the delivery of chemotherapy resistance. Overrepresented CD44 in A/Exo was confirmed under immune electron microscopy, and the levels of CD44 in exosomes were further detected by western blot. The results indicated that the protein level of CD44 was significantly upregulated in A/Exo compared with that in S/Exo (Figures 2C,D). Interestingly, the increased level of CD44 in A/exo (14.76-fold) was much higher than that in MCF-7/ADR cells (6.56-fold) (Figure 2E). These results suggest that exosomes derived from MCF-7/ADR cells contain concentrated CD44, which may be related to the DOX resistance in breast cancer cells.

### Expression of CD44 in MCF-7 After Incubation With ADR/Exo

**Figure 3:**
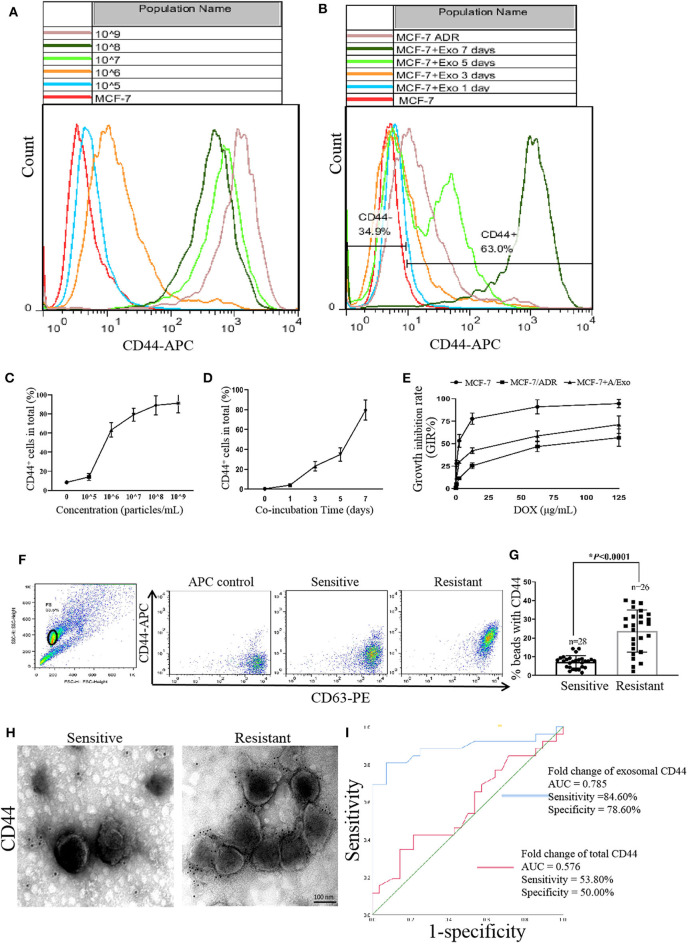
CD44 was transmitted from MCF-7/ADR to MCF-7 cells via exosomes. **(A,C)** A/Exo coincubation increased the percentage of CD44 in MCF-7 cells in a concentration-dependent manner. The expression of CD44 in MCF-7+A/Exo with 1 × 10^5^, 1 × 10^6^, 1 × 10^7^, 1 × 10^8^, and 1 × 10^9^ particles/mL was 5.73 ± 0.10%, 14.10 ± 0.70%, 63.00 ± 5.60%, 88.12 ± 9.70%, and 92.0 ± 11.20% respectively. There were no significant differences between the 1 × 10^8^ and 1 × 10^9^ group (*p* > 0.05). **(B,D)** A/Exo coincubation increased the percentage of CD44 in MCF-7 cells in a time-dependent manner. The expression of CD44 in MCF-7+A/Exo at days 0, 1, 3, 5, and 7 was 4.35 ± 0.71%, 11.42 ± 1.60%, 33.21 ± 4.70%, 58.90 ± 6.54%, and 91.10 ± 9.66% respectively. After 7 days of incubation, the expression of CD44 in MCF-7+A/Exo reached 91.10 ± 9.66%, while the expression of CD44 in MCF-7/ADR was only 77.30 ± 10.12%. **(E)** The DOX sensitivity of MCF-7 was significantly decreased after 7 days of incubation with 1 × 10^8^ particles/mL of A/Exo. **(F)** Exosomes derived from peripheral donor blood were pulled down on beads, stained with anti-CD63-PE and anti-CD44-APC, and analyzed by flow cytometry. **(G)** Quantitative analysis of flow cytometry results showed that the exosomal CD44 in the serum of nonresponders was significantly higher than that in chemotherapy-responsive group (*p* < 0.0001). “*n*” stands for the number of patients. **(H)** Immunogold TEM results showed that exosomal CD44 in the nonresponders was significantly higher than that in the chemotherapy-responsive group. **(I)** ROC curves indicated that exosomal CD44 shows AUC = 0.785, a sensitivity of 84.60%, and a specificity of 78.60%, while serum CD44 shows an AUC of 0.576, a sensitivity of 53.80%, and a specificity of 50.00%.

To further evaluate the expression of CD44 in MCF-7 after incubation with A/Exo, we detected the CD44 expression in MCF-7+A/Exo by flow cytometry analysis. As shown in Figures 3A–D, A/Exo incubation increased the expression of CD44 in a concentration-dependent (Figures 3A,C) and time-dependent manner (Figures 3B,D). The expression of CD44 in MCF-7+A/Exo with 1 × 10^5^, 1 × 10^6^, 1 × 10^7^, 1 × 10^8^, and 1 × 10^9^ particles/mL was 5.73 ± 0.10%, 14.10 ± 0.70%, 63.00 ± 5.60%, 88.12 ± 9.70%, and 92.0 ± 11.20%, respectively after incubation for 7 days. There was no significant difference between the 1 × 10^8^ and 1 × 10^9^ group (*p* > 0.05), so 1 × 10^8^ particles/mL were selected for the following study. The expression of CD44 in MCF-7+A/Exo at days 0, 1, 3, 5, and 7 was 4.35 ± 0.71%, 11.42 ± 1.60%, 33.21 ± 4.70%, 58.90 ± 6.54%, and 91.10 ± 9.66%, respectively. The results showed that after incubation with A/Exo, there was a significant increase of the CD44 expression (*p* < 0.01). After 7 days incubation, the expression of CD44 in MCF-7+A/Exo reached 91.10 ± 9.66%, while the expression of CD44 in MCF-7/ADR was only 77.30 ± 10.12%. The DOX sensitivity of MCF-7 was significantly decreased after incubation with 1 × 10^8^ particles/mL A/Exo (Figure 3E; *p* < 0.05).

### Higher Levels of Exosomal CD44 in Nonresponsive Patients

To explore the predictive value of CD44 about chemoresistance in breast cancer patients, we assessed the levels of exosomal CD44 in serum samples by immunoelectron microscopy and flow cytometry analysis. The results showed that exosomal CD44 in the serum of nonresponders (PD and SD patients) was significantly higher than that in chemotherapy responded group (PR and CR patients) (*p* < 0.0001, Figures 3F,G). Immunogold TEM was used to further confirm the specific CD44 expression. The results were consistent with the flow cytometry analysis results (Figure 3H). Although limited, these data support the notion that the upregulated CD44 in the circulating exosomes of patients had an essential role in mediating chemotherapy resistance. ROC curves indicated that exosomal CD44 shows AUC = 0.785, a sensitivity of 84.60% and a specificity of 78.60%, while serum CD44 shows an AUC of 0.576, a sensitivity of 53.80% and a specificity of 50.00% (Figure 3I).

### Isolation and Characterization of iRGD-Exos

**Figure 4:**
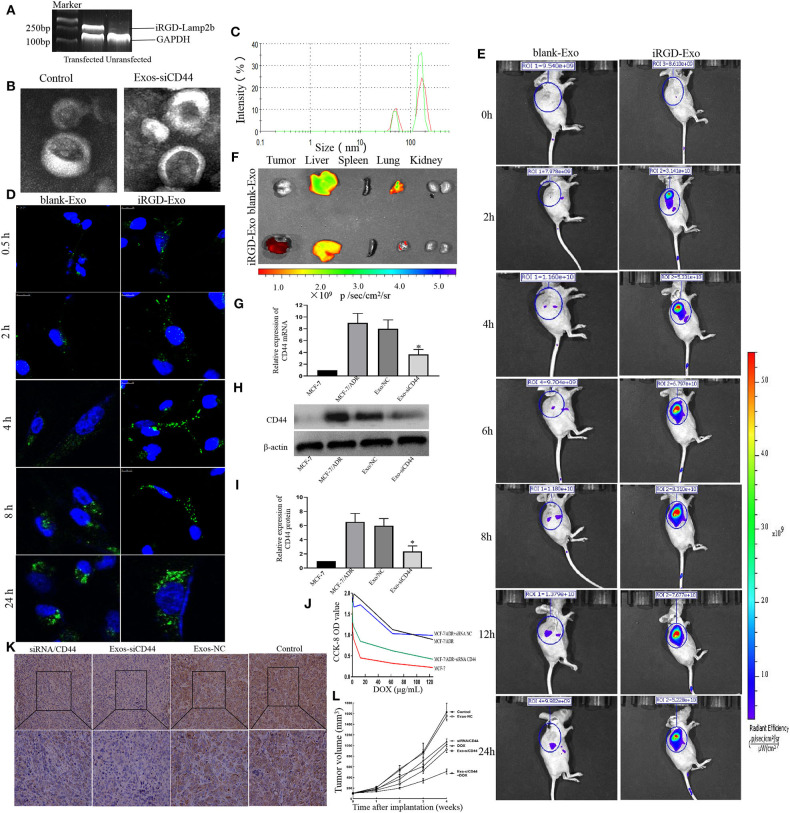
Cellular uptake and *in vivo* biodistribution of the modified exosomes. **(A)** Characterization of iRGD-Exos. The iRGD-Lamp2b mRNA expression in the MCF-7 cells was detected using RT-PCR at 48 h after transfection, and the transfected MCF-7 cells expressed high levels of iRGD-Lamp2b mRNA compared to the control group. **(B)** TEM images showed that there were no significant changes in the morphological integrity after electroporation compared with the untreated exosomes. **(C)** NTA also showed that there was no significant difference in size compared with the untreated exosomes (*p* > 0.05). **(D)** Confocal images exhibited that the early uptake efficiency was higher in the presence of iRGD-Exo than that in the presence of blank-Exo by MFI, especially at 4 h, 79.5 ± 11.2% vs. 47.3% ± 8.6. **(E)** The *in vivo* biodistribution imaging showed that iRGD-Exo could efficiently and quickly target tumor cells. At the tumor sites, the signal of the iRGD-Exo group could be detected just at 0.5 h and peaked at 8 h after injection. **(F)** Fluorescence intensity of DiD-labeled exosome in tissues. The results showed that the iRGD-Lamp2b modification facilitated the Exo targeting; the Exo was preferentially taken up by tumor cells, but also the liver and, less efficiently, lung cells. **(G)** The transferred CD44 siRNA by iRGD-Exo suffices for a significant reduction of CD44 expression in MCF-7/ADR cells. The mRNA expression of CD44 in the iRGD-Exos-siRNA/CD44 group was significantly reduced compared with the Exos-NC group and untreated MCF-7/ADR group (*p* < 0.05). **(H,I)** Western blot results showed that CD44 was reduced markedly in iRGD-Exos-siRNA/CD44 group (*p* < 0.05). **(J)** CCK-8 cell proliferation assay revealed that the optical density value (OD) of the Exo-siCD44 group was significantly reduced in comparison with that of the Exos-NC group, 0.420 ± 0.020 vs. 0.992 ± 0.103, *p* < 0.01; the Exos-NC group and untreated MCF-7/ADR cells showed no significant difference. **(K)** Immunohistochemistry results showed that the expression of CD44 was significantly decreased in the iRGD-Exos-siRNA/CD44 group compared with other groups. **(L)** Tumor volumes data indicated that the antitumor efficacy of iRGD-Exos-siRNA/CD44 was much higher than in other groups. *p < 0.05.

The tumor-targeting capability of exosomes was conferred by establishing an MCF-7 cell expressing Lamp2b, a well-characterized exosomal membrane protein, fused with iRGD targeting peptide for av integrin. To generate iRGD-positive exosomes, we fused the iRGD peptide to the extra-exosomal N terminus of murine Lamp2b protein by introducing the iRGD-Lamp2b plasmid into MCF-7 cells. The iRGD-Lamp2b mRNA expression in the MCF-7 cells was detected using RT-PCR at 48 h after transfection; the transfected MCF-7 cells expressed high levels of iRGD-Lamp2b mRNA compared to control group (Figure 4A). The purified exosomes were then electroplated, and the Exos-siCD44 was obtained. The typical structures of exosomes were observed by TEM, and there were no significant changes in the morphological integrity after electroporation compared with the untreated ones (Figure 4B, *p* > 0.05). NTA also showed that the particle size distribution was consistent between Exos-siCD44 and the untreated exosomes (Figure 4C, *p* > 0.05). The aforementioned results indicated that the modification and electroporation did not affect the morphology of exosomes.

The levels of CD44 siRNA in exosomes were assayed by an LNA primer-based quantitative RT-PCR assay; the final concentration of CD44 siRNA was ~0.152 pmol/μg. The results clearly showed that CD44 siRNA can be effectively packaged into exosomes.

### Targeting of iRGD-Exos *in vitro* and *in vivo*

To investigate the intracellular uptake and the targeting ability of iRGD-Exos for breast cancer cells *in vitro*, the Alexa Fluor 488-CD63 labeled exosomes were cultured with MCF-7 cells. Confocal microscopy was used to observe the accumulation of CD63-labeled exosomes in MCF-7 cells (Figure 4D). Higher signals were observed from iRGD-Exo compared to blank-Exo at 0.5, 2, 4, 8, and 24 h after treatment, respectively. The early uptake efficiency was higher in the presence of iRGD-Exo than that in the presence of blank-Exo (especially at 4 h, 79.5 ± 11.2% vs. 47.3% ± 8.6, *p* < 0.05). However, the difference was not significant at 24 h (87.5 ± 10.33% vs. 91.4% ± 9.5, *p* > 0.05).

To further determine the targeting ability of iRGD-Exos for breast cancer cells *in vivo*, DiD-labeled iRGD-Exo were intravenously injected into tail veins of MCF-7/ADR xenograft mice. Animals were examined using bioimaging system at 0, 2, 4, 6, 8, 12, and 24 h after injection (Figure 4E). In the DiD-labeled iRGD-Exo group, the signal was detected 0.5 h after injection and peaked after 8 h. The signal was mainly detected in the tumor sites, while some low signals were detected in the liver and lungs. Contrary, in the blank-Exo group, low signal was detected in the tumor site while the higher signal was detected in the liver and lung (Figure 4F). The aforementioned results showed that iRGD-Exo was tumor specific.

### siRNA Silences CD44 Expression in MCF-7/ADR Cells

Next, we modified the MCF-7-derived exosomes loaded with siRNA against CD44 to observe the effects of targeting reduced CD44 expression in luminal A breast cancer cells. MCF-7/ADR cells were incubated with iRGD-Exos-siRNA/CD44 (Exos-siCD44) and Negative Control siRNA (Exos-NC). To validate the suppression of CD44 in MCF-7/ADR cells by Exos-siCD44, qRT-PCR and western blot were preformed 48 h after treatment. As shown in Figures 4G–I, the expression of CD44 in Exo-siCD44 group was significantly reduced compared with the Exos-NC group and untreated MCF-7/ADR group (*p* < 0.05). This indicated that Exos-siCD44 could effectively silence CD44 expression in MCF-7/ADR cells.

### iRGD-Exosomes-siRNA CD44 Increased Chemosensitivity in MCF-7/ADR Cells

CCK-8 cell proliferation assay revealed that the optical density value (OD) of Exo-siCD44 group was significantly reduced in comparison with that of the Exos-NC group (0.420 ± 0.020 vs. 0.992 ± 0.103, *p* < 0.01) (Figure 4J). There was no significant difference between the Exos-NC group, and the untreated MCF-7/ADR group (0.892 ± 0.125, *p* > 0.05) showed no significant difference. These results indicated that Exo-siCD44 increased DOX sensitivity in MCF-7/ADR cells without significant cytotoxicity.

### *In vivo* Antitumor Efficacy of iRGD-Exos-siRNA/CD44

Next, we assessed the antitumor effect of the Exo-siCD44 in tumor-bearing mice. As shown in Figure 4L, the PBS-treated tumors increased 16.32-fold in volume. The DOX and free siRNA/CD44 alone treated tumors showed a 9.51-fold and 11.42-fold increase, whereas the Exo-siCD44 treated tumors showed an 8.84-fold increase after 21 days of treatment. DOX and Exo-siCD44 cotreatment showed a 4.12-fold increase, and there were no significant changes in the Exo-NC group compared with the PBS-treated group. No mortality occurred, suggesting that Exo-siCD44 was not toxic to the animals. Notably, the expression of CD44 was significantly decreased in the Exo-siCD44 group compared with siRNA/CD44 group (Figure 4K).

## Discussion

Drug resistance has severely limited the clinical efficacy of chemotherapy in patients with breast cancer. Previous studies have shown that the exosomes, as vectors of signaling molecules, could alter the chemotherapeutic sensitivity of receptor cells by intercellular communication (17, 18). Exosomes, secreted from tumor cells, can remodel tumor environment by promoting tumor metastasis and multidrug resistance through direct drug export, transport of drug efflux pumps, and miRNAs exchange among cells. Although genomic and transcriptomic profiles of exosomes associated with drug resistance have already been established (19–21), exosomal proteomics in drug resistance have rarely been investigated. In this work, characterizing of the exosomal protein components from drug-resistant cells furthered our understanding on their role in the progression of chemoresistance transmission.

In this study, we firstly examined the effects of DOX on the release and biological function changes of exosomes. Then proteomics was employed to profile the intracellular information and signal pathways mediated by exosomes. These identified differential proteins were helpful to illustrate the mechanisms of drug resistance transmission between MCF-7/ADR and MCF-7 cells. Our results indicate that chemotherapy-resistant tumor cells selectively shuttled their secreted specific proteins into exosomes and that exosomes transported and delivered these proteins to other tumor cells. Two hundred and fifty differentially expressed proteins were found in ADR/Exo compared with S/Exo. The higher level of chemotherapy-resistant proteins ABCB1, CD44, GSTP1, PAICS, AXL, ANXA2, HSPB1, ICAM1, and MMP-1 etc. and the lower levels of LOX, HIC2, HRG, COL1A1, HTRA1, AEBP1, and MAP2K5 proteins that related with the enhanced drug sensitivity and growth suppression of cancer cells were concentrated in ADR/Exo. The exosome proteomics data as an important complement to previous genomics and transcriptomics studies played an important role in the study of drug resistance transmission.

In the previous study, we have found that the percentage of CD44+ cells in the MCF-7 cell line is extremely low. Interestingly, we also found that DOX-induced the secretion of exosomes and increased the encapsulation of CD44 in exosomes in MCF-7/ADR cells; yet, the mechanism of enrichment remains unclear. CD44, as the receptor for hyaluronic acid (HA), mediates intracellular cell signaling through its interaction with cytoskeletal proteins and is involved in cell adhesion, migration, drug resistance, and signal transmission in some drug-resistant cancer cell lines (22–25). Recent studies have demonstrated that the up-regulation of CD44 in NSCLC cells can significantly reduce the cisplatin sensitivity of chemoresistant NSCLC cells (26). Harshman et al. (27) found that CD44 was highly enriched in exosomes of drug-resistant multiple myeloma cell and mediated resistance transmission by initiating intracellular signaling pathways. Our data further indicated that exosomes of DOX-resistant cells could enrich and transfer resistance properties to DOX-sensitive counterparts, especially the CD44; the difference level of exosomal-CD44 was significantly higher than that in cells. However, the mechanism of selective enrichment of exosomes still remains unknown. Interestingly, coincubationwith Exo-siCD44 significantly increased sensitivity to chemotherapy in MCF-7/ADR cells and tumor-bearing mice, thus suggesting that exosomal CD44 have an important role for the MCF-7 cells that acquired DOX resistance. Therefore, it is important to elucidate the role of CD44 in the development of chemoresistant and develop effective therapeutic strategies to overcome chemoresistance.

In this study, we used MCF-7 cells as a source of exosomes (S/Exo). Cancer-derived exosomes have been reported to be used as a delivery platform that could confer cancer cell tropism-dependent targeting (28). The tumor-targeting capability of S/Exo was further improved by expressing exosomal membrane protein-Lamp2b, which was fused with the av integrin targeting peptide-iRGD. We established the delivery vehicle using tumor-derived exosomes loaded with siRNA against CD44 to disrupt the CD44 expression of MCF-7/ADR cells. Our results demonstrated that iRGD-Exos could effectively target tumor cells *in vitro* and *in vivo*. Moreover, iRGD-Exos loaded with siRNA/CD44 inhibited the cell proliferation *in vitro* and tumor growth *in vivo*. The targeting modified exosomes used as a carrier, can rapidly, efficiently, and specifically deliver the therapeutic gene to breast cancer cells, and reduce the aggregation of nontarget sites. Our study provides a broader prospect for clinical gene therapy and has important clinical implications for breast cancer chemotherapy.

## Conclusions

This study confirmed that DOX-resistant breast cancer cells spread resistance capacity to sensitive cells by releasing exosomes carrying resistance-associated proteins. Exosomes act as a double-edged sword while transmitting drug resistance information; they can also efficiently target tumor cells to play the role of therapeutic vectors. Exosomes modified with iRGD-loading siRNA could efficiently target MCF-7/ADR breast cancer cells and reverse the phenotype to improve therapeutic response.

## Data Availability Statement

The raw data supporting the conclusions of this article will be made available by the authors, without undue reservation, to any qualified researcher.

## Ethics Statement

The studies involving human participants were reviewed and approved by Medical Research Ethics Committee of Binzhou Medical University Hospital (Approval No. LW-012). The patients/participants provided their written informed consent to participate in this study. The animal study was reviewed and approved by Institutional Animal Care and Use Committee of Binzhou Medical University Hospital (No. SYXK 20180022).

## Author Contributions

XW designed the experiments, performed the experiment, analyzed results, and wrote the manuscript. KC, GZ, ZJ, YY, JG, YH, and FG performed the experiments and analyzed the results. XL, WZ, and HS summarized the clinical data and performed the statistical analysis. JD revised the manuscript. ZY designed the experiments and revised the manuscript. All authors contributed to the article and approved the submitted version.

## Conflict of Interest

The authors declare that the research was conducted in the absence of any commercial or financial relationships that could be construed as a potential conflict of interest.

## Abbreviations

A/Exo: exosomes extracted from MCF-7/ADR cells
APC: allophycocyanin
BSA: bovine serum albumin
CR: complete response
CCK-8: Cell Counting Kit-8
DEPs: differently expressed proteins
DOX: doxorubicin
ELISA: enzyme-linked immunosorbent assay
FBS: fetal bovine serum
GO analysis: gene ontology analysis
KEGG: Kyoto Encyclopedia of Genes and Genomes
kV: kilovolts
LC-MS/MS: liquid chromatography linked to tandem mass spectrometry
MCF-7/ADR: doxorubicin resistance MCF-7 cells lines reduced by adriamycin
MDR: multidrug resistance
NTA: ZetaView nanoparticle-tracking analysis
PBS: phosphate-buffered saline
PD: progressive disease
PE: phycoerythrin
PR: partial response
RECIST: Response Evaluation Criteria in Solid Tumors
RT-PCR: Real-time-polymerase chain reaction
SD: stable disease
S/Exo: exosomes extracted from MCF-7 cells
TEM: transmission electron microscopy
TMT™: Tandem Mass Tag.
