# Supplementary material for: Enrichment of CD44 in Exosomes From Breast Cancer Cells Treated With Doxorubicin Promotes Chemoresistance
Source: Front Oncol. 2020 Jul 14;10:960. doi: 10.3389/fonc.2020.00960 (PMC7373100; doi:10.3389/fonc.2020.00960)
Supplement: Supplementary file 1 [file Data_Sheet_1.docx]

Supplementary Material

## Supplementary Figure 1

##
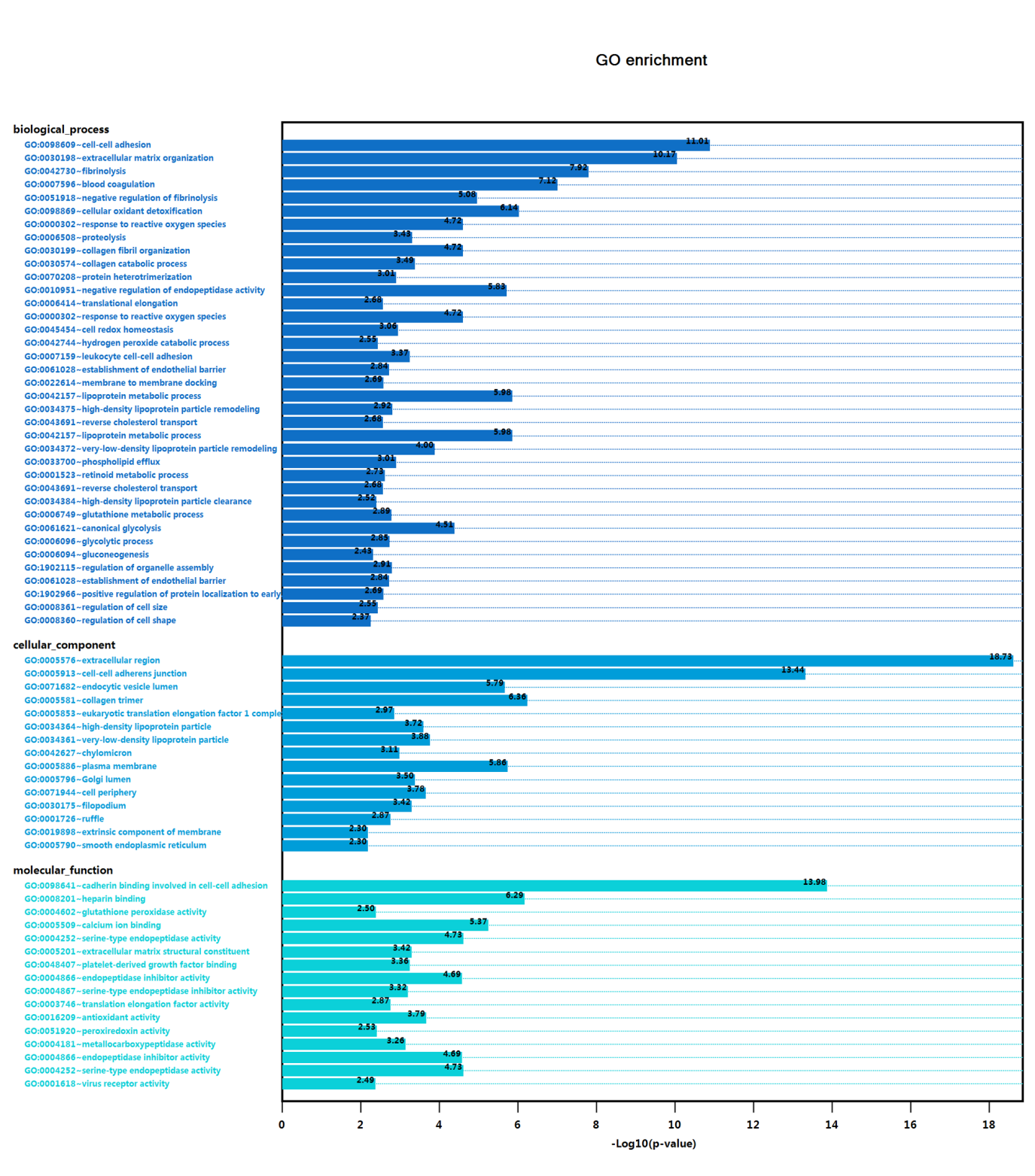


**Supplementary Figure 1** GO analysis of differently expressed proteins. The differently expressed proteins mainly regulate cell-cell adhesion, cell-cell adherens junction, extracellular matrix organization, extrcellular region, and cadherin binding involved in cell-cell adhesion.

## Supplementary Table 1

**Table 1 Down-regulated proteins in ADR/Exo (log2FoldChange|≧1.0)**

| **ID** | **geneName** | **log2FC** | **p.value** | **FDR** |
| --- | --- | --- | --- | --- |
| Q8N139 | ABCA6 | -2.749913623 | 1.01E-20 | 1.27E-18 |
| Q6UXH8 | CCBE1 | -2.568773782 | 7.06E-15 | 4.20E-14 |
| Q15113 | PCOLCE | -2.030636749 | 4.16E-20 | 3.02E-18 |
| P42226 | STAT6 | -2.029372498 | 1.31E-17 | 1.75E-16 |
| P28300 | LOX | -1.964732595 | 2.11E-17 | 2.59E-16 |
| Q92616 | GCN1 | -1.90252404 | 1.52E-12 | 5.61E-12 |
| Q96JB1 | DNAH8 | -1.74259009 | 2.20E-16 | 1.99E-15 |
| P45877 | PPIC | -1.733721336 | 9.79E-17 | 9.92E-16 |
| Q96JB3 | HIC2 | -1.641697244 | 4.05E-17 | 4.46E-16 |
| Q9P2B2 | PTGFRN | -1.641418313 | 8.64E-19 | 2.11E-17 |
| P04196 | HRG | -1.614183536 | 6.78E-19 | 1.81E-17 |
| P09104 | ENO2 | -1.608579238 | 3.88E-17 | 4.32E-16 |
| P02452 | COL1A1 | -1.540018335 | 2.26E-19 | 8.64E-18 |
| Q9H444 | CHMP4B | -1.458281503 | 3.59E-17 | 4.10E-16 |
| P69891 | HBG1 | -1.410841247 | 2.43E-19 | 8.91E-18 |
| Q92743 | HTRA1 | -1.407445068 | 2.32E-17 | 2.80E-16 |
| P35527 | KRT9 | -1.286180149 | 1.39E-16 | 1.32E-15 |
| P35442 | THBS2 | -1.281549857 | 4.80E-20 | 3.02E-18 |
| Q8WUM4 | PDCD6IP | -1.272078017 | 3.80E-21 | 6.70E-19 |
| P04264 | KRT1 | -1.255475836 | 1.00E-17 | 1.47E-16 |
| Q86UQ4 | ABCA13 | -1.235719556 | 4.95E-16 | 3.96E-15 |
| P98095 | FBLN2 | -1.234047063 | 2.53E-18 | 5.02E-17 |
| O00560 | SDCBP | -1.228518401 | 1.16E-17 | 1.65E-16 |
| O75787 | ATP6AP2 | -1.217030018 | 4.58E-16 | 3.74E-15 |
| Q14644 | RASA3 | -1.197061891 | 3.46E-17 | 4.02E-16 |
| P05543 | SERPINA7 | -1.194182102 | 3.87E-15 | 2.54E-14 |
| P27169 | PON1 | -1.183708286 | 1.42E-15 | 1.03E-14 |
| Q96IY4 | CPB2 | -1.18209426 | 5.65E-15 | 3.52E-14 |
| Q8IUX7 | AEBP1 | -1.177460612 | 5.67E-15 | 3.52E-14 |
| Q13163 | MAP2K5 | -1.166608274 | 7.20E-22 | 2.11E-19 |
| Q9NQ79 | CRTAC1 | -1.153827418 | 4.49E-12 | 1.47E-11 |
| Q9UBX5 | FBLN5 | -1.150018759 | 2.87E-18 | 5.33E-17 |
| P22352 | GPX3 | -1.14279763 | 1.14E-15 | 8.60E-15 |
| P08123 | COL1A2 | -1.12703972 | 5.74E-18 | 9.21E-17 |
| P00734 | F2 | -1.123334375 | 6.85E-20 | 3.55E-18 |
| P35908 | KRT2 | -1.118460938 | 1.14E-17 | 1.65E-16 |
| P13645 | KRT10 | -1.103906127 | 2.02E-15 | 1.39E-14 |
| P12110 | COL6A2 | -1.099515524 | 1.10E-14 | 6.23E-14 |
| Q08830 | FGL1 | -1.089668388 | 2.36E-17 | 2.81E-16 |
| Q03181 | PPARD | -1.085861659 | 3.87E-16 | 3.22E-15 |
| P51888 | PRELP | -1.081786073 | 4.83E-12 | 1.57E-11 |
| Q9UKL3 | CASP8AP2 | -1.062406911 | 2.12E-14 | 1.14E-13 |

## Supplementary Table 2

**Table 2 Up-regulated proteins in ADR/Exo (log2FoldChange|≧1.0)**

| **ID** | **geneName** | **log2FC** | **p.value** | **FDR** |
| --- | --- | --- | --- | --- |
| P21589 | NT5E | 1.009819327 | 4.74E-14 | 2.35E-13 |
| P63313 | TMSB10 | 1.010447833 | 1.98E-13 | 8.62E-13 |
| A0A0C4DH25 | IGKV3D-20 | 1.01148149 | 8.14E-16 | 6.29E-15 |
| P30530 | AXL | 1.019494342 | 1.02E-10 | 2.69E-10 |
| P36873 | PPP1CC | 1.020861041 | 2.07E-06 | 3.15E-06 |
| Q6Y7W6 | GIGYF2 | 1.054584991 | 0.008236081 | 0.009792156 |
| Q01469 | FABP5 | 1.082601951 | 1.33E-12 | 4.99E-12 |
| P07355 | ANXA2 | 1.085642302 | 5.75E-18 | 9.21E-17 |
| P05783 | KRT18 | 1.089381553 | 3.56E-19 | 1.16E-17 |
| P15311 | EZR | 1.092422023 | 1.16E-16 | 1.16E-15 |
| P08183 | ABCB1 | 1.100920565 | 1.80E-13 | 7.88E-13 |
| P03956 | MMP1 | 1.107720918 | 3.41E-16 | 2.89E-15 |
| P29279 | CTGF | 1.111400029 | 3.13E-19 | 1.10E-17 |
| P11234 | RALB | 1.132346931 | 4.82E-10 | 1.15E-09 |
| P05362 | ICAM1 | 1.136468428 | 5.81E-20 | 3.41E-18 |
| O43707 | ACTN4 | 1.137934567 | 1.69E-13 | 7.44E-13 |
| Q9NZI8 | IGF2BP1 | 1.137949597 | 1.06E-14 | 6.11E-14 |
| P62318 | SNRPD3 | 1.154095566 | 1.78E-17 | 2.28E-16 |
| Q9BXJ9 | NAA15 | 1.160094638 | 3.20E-10 | 7.89E-10 |
| Q02952 | AKAP12 | 1.165918327 | 3.42E-14 | 1.73E-13 |
| Q14166 | TTLL12 | 1.211203879 | 5.86E-17 | 6.22E-16 |
| P13987 | CD59 | 1.222304607 | 3.22E-23 | 1.42E-20 |
| Q9HC35 | EML4 | 1.232950225 | 5.39E-17 | 5.79E-16 |
| P09936 | UCHL1 | 1.254060267 | 1.28E-17 | 1.75E-16 |
| P0DP24 | CALM2 | 1.256303687 | 5.77E-15 | 3.53E-14 |
| Q05639 | EEF1A2 | 1.257059569 | 1.04E-09 | 2.32E-09 |
| Q96KK5 | HIST1H2AH | 1.259356299 | 2.97E-14 | 1.54E-13 |
| P31947 | SFN | 1.284978449 | 1.24E-16 | 1.22E-15 |
| P01705 | IGLV2-23 | 1.292466271 | 2.15E-21 | 4.75E-19 |
| P04792 | HSPB1 | 1.329895601 | 2.52E-18 | 5.02E-17 |
| Q15485 | FCN2 | 1.334237769 | 4.60E-19 | 1.45E-17 |
| P80723 | BASP1 | 1.34673813 | 2.91E-13 | 1.23E-12 |
| P00739 | HPR | 1.376022578 | 2.91E-18 | 5.33E-17 |
| P17813 | ENG | 1.406909301 | 6.51E-20 | 3.55E-18 |
| P16070 | CD44 | 1.507068654 | 7.13E-18 | 1.09E-16 |
| P48509 | CD151 | 1.511050753 | 9.36E-18 | 1.40E-16 |
| Q9GZP8 | IMUP | 1.512574238 | 8.84E-21 | 1.27E-18 |
| O95816 | BAG2 | 1.554351074 | 2.29E-18 | 4.81E-17 |
| P49006 | MARCKSL1 | 1.574548865 | 2.00E-16 | 1.85E-15 |
| Q16186 | ADRM1 | 1.70465692 | 3.88E-09 | 8.03E-09 |
| P31431 | SDC4 | 1.852031808 | 1.52E-18 | 3.51E-17 |
| P21926 | CD9 | 1.948493353 | 7.11E-17 | 7.37E-16 |
| P35613 | BSG | 2.103885514 | 5.82E-16 | 4.62E-15 |
| P02795 | MT2A | 2.654815629 | 1.01E-13 | 4.68E-13 |

## Supplementary Table 3

**Table 3 KEGG analysis**

| **KEGG pathway** | **Proteins** |
| --- | --- |
| ECM-receptor interaction | THBS2, COL6A2, COL5A1, COL1A1, COL1A2, COL2A1, ITGB1, ITGB5, TNC, SDC4, VTN, HSPG2, CD44, CD47 |
| Focal adhesion | THBS2, COL6A2, PPP1CC, ITGB1, COL5A1, COL1A1, COL1A2, PPP1CA, FLNC, ACTN1, ITGB5, TNC, COL2A1, ACTG1, VTN, ACTN4 |
| PI3K-Akt signaling pathway | THBS2, ITGB5, TNC, GNB1, COL6A2, COL5A1, ITGB1, COL2A1, COL1A1, VTN, COL1A2 |
| Complement and coagulation cascades | C5, CD59, SERPIND1, FGB, F13A1, F5, F10, F9, MBL2, SERPINF2, C3, F2, PLG, CPB2 |
| Platelet activation | COL5A1, ITGB1, PPP1CC, COL2A1, FGB, COL1A1, ACTG1, PLCB3, COL1A2, PPP1CA |
| Amoebiasis | ACTN1, COL5A1, COL2A1, COL1A1, PLCB3, ACTN4, HSPB1, COL1A2 |
| Protein digestion and absorption | CPA2, COL6A2, COL5A1, COL2A1, COL1A1, COL1A2, CPB2 |
| Leukocyte transendothelial migration | ACTN1, EZR, ITGB1, ICAM1, MSN, ACTG1, ACTN4 |
| PPAR signaling pathway | FABP5, UBC, LPL, PPARD, APOA1, PLTP, MMP1 |
| Biosynthesis of amino acids | ALDOB, TPI1, GAPDH, PFKP, IDH1, ENO2, PGAM2 |
| Glycolysis / Gluconeogenesis | ALDOB, TPI1, GAPDH, PFKP, ENO2, PGAM2 |
| Carbon metabolism | ALDOB, TPI1, GAPDH, PFKP, IDH1, ENO2, PGAM2 |
| Biosynthesis of antibiotics | ALDOB, UAP1, TPI1, GAPDH, PCYOX1, PAICS, PFKP, IDH1, ENO2, PGAM2 |
| Fructose and mannose metabolism | ALDOB, TPI1, PFKP |
| Metabolic pathways | PTGES3, ALDOB, UAP1, TPI1, GAPDH, PFKP, NT5E, CKM, PLCB3, AMY1A, PRDX6, GSS, PAICS, IDH1, ENO2, PGAM2, ANPEP |
| Cell adhesion molecules (CAMs) | ICOSLG, ITGB1, ICAM1, SDC4, NCAM1 |
| Protein processing in endoplasmic reticulum | HSPA8, BAG2, HSPA5, HYOU1, ERP29 |
| Adherens junction | IQGAP1, ACTN1, [ACTG1](https://www.genecards.org/cgi-bin/carddisp.pl?gene=ACTG1&keywords=P63261), CSNK2B, ACTN4 |
| Protein processing in endoplasmic reticulum | HSPA8, BAG2, HSPA5, HYOU1, [ACTG1](https://www.genecards.org/cgi-bin/carddisp.pl?gene=ACTG1&keywords=P63261) |
| Staphylococcus aureus infection | [C5](https://www.genecards.org/cgi-bin/carddisp.pl?gene=C5&keywords=P01031), MBL2,[ICAM1](https://www.genecards.org/cgi-bin/carddisp.pl?gene=ICAM1&keywords=P05362), [C3](https://www.genecards.org/cgi-bin/carddisp.pl?gene=C3&keywords=P01024), PLG |
| ABC transporters | ABCA6, ABCB1, ABCA13 |
| Systemic lupus erythematosus | ACTN1, C5, HIST1H2BL, SNRPD3, HIST1H3A, HIST1H2AH, ACTN4, C3, HIST1H4A |
| Alcoholism | HIST1H2BL, GNB1, CALM2, PPP1CC, HIST1H3A, HIST1H2AH, PPP1CA, HIST1H4A |
| Phagosome | THBS2, ITGB5, CORO1A, MBL2, ITGB1, TUBB2B, ACTG1, C3 |
| Arrhythmogenic right ventricular cardiomyopathy | ITGB5, ITGB1, ACTG1 |
| Hypertrophic cardiomyopathy | ITGB5, ITGB1, ACTG1 |
| Dilated cardiomyopathy | ITGB5, ITGB1, ACTG1 |
| Dopaminergic synapse | GNB1, CALM2, PPP1CC, PLCB3, PPP1CA |
| Circadian entrainment | GNB1, CALM2, PLCB3 |
| Oxytocin signaling pathway | CALM2, PPP1CC, MYL6, ACTG1, PLCB3, MAP2K5, PPP1CA |
| Vascular smooth muscle contraction | CALM2, PPP1CC, MYL6, PLCB3, PRKCE, PPP1CA |
| Inflammatory mediator regulation of TRP channels | CALM2, PPP1CC, PLCB3, PRKCE, PPP1CA |
| Long-term potentiation | CALM2, PPP1CC, PLCB3, PPP1CA |
| Dopaminergic synapse | GNB1, CALM2, PPP1CC, PLCB3, PPP1CA |
| Amphetamine addiction | CALM2, PPP1CC, PPP1CA |
| cGMP-PKG signaling pathway | CALM2, PPP1CC, PLCB3, PRKCE, PPP1CA |
| Aldosterone synthesis and secretion | CALM2, PLCB3, PRKCE |
| Adrenergic signaling in cardiomyocytes | CALM2, PPP1CC, PLCB3, PPP1CA |
| Insulin resistance | PPP1CC, Q02156, PPP1CA |
| Oocyte meiosis | CALM2, PPP1CC, PPP1CA |
| Insulin signaling pathway | CALM2, PPP1CC, PPP1CA |
| cAMP signaling pathway | CALM2, PPP1CC, PPP1CA |

## Supplementary Table 4

**Table 4 Antibodies for** **Flow cytometry analysis**

| **Protein** | **Antibody** |
| --- | --- |
| ABCA6 | Abcam, Anti-ABCA6 antibody, CN. ab180567 |
| CCBE1 | Invitrogen, CCBE1 Polyclonal Antibody, CN.PA5-59534 |
| PCOLCE | Abcam, Anti-PCOLCE antibody, CN. ab220040 |
| STAT6 | BioLegend, [PE anti-STAT6 Phospho (Tyr641) Antibody](https://www.biolegend.com/en-us/products/pe-anti-stat6-phospho-tyr641-antibody-13553), CN. 686003 |
| LOX | BioLegend, APC anti-human LOX-1 Antibody, CN. 358605 |
| GCN1 | Abcam, Anti-GCN1 antibody, CN. ab86139 |
| DNAH8 | Abcam, Anti-DNAH8 antibody, CN. ab121989 |
| PPIC | Abcam, Anti-PPIC antibody [EPR15355], CN. ab184552 |
| HIC2 | Proteintech, HIC2 Rabbit Polyclonal antibody, CN. 22788-1-AP |
| PTGFRN | Abcam, Anti-PTGFRN antibody, CN. ab97567 |
| HRG | Proteintech, HRG Rabbit Polyclonal antibody, CN. 26252-1-AP |
| ENO2 | BioLegend, Alexa Fluor® 488 anti-NSE Antibody, CN. 804904 |
| COL1A1 | Abcam, Anti-Collagen I antibody [EPR22894-89], CN. ab260043 |
| CD44 | BioLegend, APC anti-human CD44 Antibody, CN. 338806 |
| CD151 | BioLegend, APC anti-human CD151 (PETA-3) Antibody, CN. 350405 |
| IMUP | Abcam, Anti-IMUP antibody, CN. ab221063 |
| BAG2 | Abcam, Anti-BAG2 antibody [EPR3568], CN. ab80596 |
| MARCKSL1 | Abcam, [Anti-MARCKS like protein antibody [EPR15353]-C-terminal, CN. ab184546](https://www.abcam.cn/marcks-like-protein-antibody-epr15353-c-terminal-ab184546.html) |
| ADRM1 | Abcam, Anti-ADRM1 antibody [EPR11449(B)] (Phycoerythrin), CN. ab211702 |
| SDC4 | RD, Human Syndecan-4 APC-conjugated Antibody, CN. FAB29181A |
| CD9 | BioLegend, APC anti-human CD9 Antibody, CN. 312107 |
| BSG | BioLegend, APC anti-human CD147(BSG)Antibody, CN. 306213 |
| MT2A | Proteintech, CoraLite488-Conjugated CES1 Antibody, CN. CL488-67079 |
| CD63 | Thermo Fisher Scientific, PE anti-human CD63 Monoclonal Antibody (H5C6), CN. 12-0639-41 |
